# Natural language processing of radiology reports to investigate the effects of the COVID-19 pandemic on the incidence and age distribution of fractures

**DOI:** 10.1007/s00256-021-03760-5

**Published:** 2021-04-13

**Authors:** Florian Jungmann, B. Kämpgen, F. Hahn, D. Wagner, P. Mildenberger, C. Düber, R. Kloeckner

**Affiliations:** 1 Department of Diagnostic and Interventional Radiology, University Medical Center of the Johannes Gutenberg-University Mainz, Langenbeckst. 1, 55131 Mainz, Germany; 2 Empolis Information Management, Kaiserslautern, Germany; 3 Department of Orthopedics and Traumatology, University Medical Center of the Johannes Gutenberg-University Mainz, Mainz, Germany

**Keywords:** Radiology, Radiological reports, Natural language processing, Fracture, Radiographs

## Abstract

**Objective:**

During the COVID-19 pandemic, the number of patients presenting in hospitals because of emergency conditions decreased. Radiology is thus confronted with the effects of the pandemic. The aim of this study was to use natural language processing (NLP) to automatically analyze the number and distribution of fractures during the pandemic and in the 5 years before the pandemic.

**Materials and methods:**

We used a pre-trained commercially available NLP engine to automatically categorize 5397 radiological reports of radiographs (hand/wrist, elbow, shoulder, ankle, knee, pelvis/hip) within a 6-week period from March to April in 2015–2020 into “fracture affirmed” or “fracture not affirmed.” The NLP engine achieved an *F*_1_ score of 0.81 compared to human annotators.

**Results:**

In 2020, we found a significant decrease of fractures in general (*p* < 0.001); the average number of fractures in 2015–2019 was 295, whereas it was 233 in 2020. In children and adolescents (*p* < 0.001), and in adults up to 65 years (*p* = 0.006), significantly fewer fractures were reported in 2020. The number of fractures in the elderly did not change (*p* = 0.15). The number of hand/wrist fractures (*p* < 0.001) and fractures of the elbow (*p* < 0.001) was significantly lower in 2020 compared with the average in the years 2015–2019.

**Conclusion:**

NLP can be used to identify relevant changes in the number of pathologies as shown here for the use case fracture detection. This may trigger root cause analysis and enable automated real-time monitoring in radiology.

## Background

The COVID-19 pandemic has led to profound changes in the health-care system. In China, elective as well as emergency procedures in orthopedic and trauma services decreased during the COVID-19 pandemic [1]. In Europe, orthopedic and trauma surgery clinics were also affected during the first weeks of the COVID-19 pandemic, with a decrease in inpatient admission and thus a decrease in revenue [2]. For radiology, this will lead to a significant decrease in orthopedic and trauma imaging. In Paris and in London, the number of hand and upper limb emergencies declined during the COVID-19 pandemic lockdown [3, 4]. The mere number of fractures did not change during the pandemic [3]. It is unclear if a decrease in hospital admissions correlates with a decrease in fractures. Analyzing the mere number of examinations for radiographs is simple, because the information can be retrieved from radiology information systems (RIS). However, to categorize these radiological examinations, for example, into “fracture” versus “no fracture,” manual evaluation of the free-text reports by medical experts is mandatory. Natural language processing (NLP) is a computer-based approach to analyzing free text [5]. NLP enables the structuring of unstructured clinical information, such as that available in most radiological free-text reports [6, 7]. Medical ontologies such as RadLex facilitate the categorization of unstructured information by using concepts with various synonyms or related terms [8]. NLP-enabled algorithms showed to be promising for automatic identification of periprosthetic femur fractures and osteoporosis-related skeletal site-specific fractures from radiological reports [9, 10]. Compared to manual annotation of radiological reports, NLP is able to automatically annotate data more efficiently.

The aim of this study was to use NLP to automatically analyze the number and distribution of fractures during the COVID-19 pandemic and in the 5 years before the pandemic. The present work aims to investigate the impact of the COVID-19 pandemic on different types of fractures in a tertiary care institution based on radiological free-text reports of radiographs.

## Methods

This retrospective, single-site, controlled study did not require professional legal advice by the Institutional Review Board or informed consent from patients, according to state hospital law. All free-text reports were fully de-identified.

### Radiological reports

We retrospectively searched for all radiological free-text reports of radiographs of the hand/wrist, elbow, shoulder, ankle, knee, and pelvis/hip between March 18 and April 30 in 2015–2020. We chose this time period because the nationwide curfew in Germany came into force on March 18 and was eased on April 30. We defined the 5-year period of 2015–2019 as the comparison group. We queried our RIS for all outpatients sent from the orthopedics and trauma clinics as well as from the pediatric clinics (including emergency room settings). Inpatients were excluded from this study, because we intended to avoid including follow-up examinations in patients with surgically treated injuries in our analysis. In total, we analyzed 5397 radiological free-text reports. For analysis, we categorized the reports into three groups based on the patient’s age (< 18 years, 18–65 years, and > 65 years). The mean age of the patients in 2015–2019 was 39.81 years (standard deviation 25.62 years), and in 2020, it was 47.97 years (standard deviation 25.41 years). In 2015–2019, the male-to-female ratio was 1.12 (m: 2506; f: 2240), whereas in 2020, it was 1.09 (m: 339; f: 312). All reports were written in German.

### Natural language processing

We used a pre-trained commercially available NLP engine to automatically categorize the radiological reports of radiographs. The Empolis Healthcare Analytics Services (HAS; Empolis Information Management GmbH) was used to analyze the anonymized reports. HAS creates an NLP pipeline into action. This pipeline consists of different steps, such as cleansing, contextualization, concept recognition using RadLex, and negation detection [7].

HAS used a neural language model and word embeddings pre-trained with the NLP library spaCy [11] on more than 100,000 radiological reports (in German). For negation detection, a deep learning approach based on NegEx [12] and spaCy [11] pre-trained on more than 2000 manually labeled reports was used.

Fine-tuning of the NLP model proved useful in previous work [7]. To minimize further efforts in manual labeling and to strictly separate training from test data, 215 randomly selected reports of 2018 were manually examined for synonyms of fracture and manually labeled with negation information by medical students at Empolis. In the present study, the results underwent binary annotation for fracture (RID4650): 1 for “fracture affirmed” and 0 for “fracture not affirmed.” We did not analyze the reports for suspected or negated fractures.

To measure the performance of the NLP engine, two medical students manually categorized all radiological free-text reports in 2019 and 2020 into “fracture affirmed” or “fracture not affirmed.” The annotators had to count only those cases with “fracture affirmed” in which the fracture was confirmed with certainty. Reports in which further investigations (CT, MRI) were recommended were not counted. For consensus reading, a board-certified radiologist reviewed those cases in which the results of annotator 1 and annotator 2 did not match. The results of these manual annotations were compared to those automatically generated by the NLP engine.

### Statistical analysis

To measure the performance of the NLP engine, we used the *F*_1_ score as an overall measure of system performance [13]. Cohen’s kappa coefficient [*κ*] was used to measure the interrater agreement between annotator 1 and annotator 2.

We analyzed the free-text reports separately for the years 2015–2020. We compared the mean number of fractures as well as the mean number of radiographs for 2015–2019 with 2020. In addition, we analyzed whether the age of the patients with affirmed fracture or the location of the fractures was different during the pandemic compared with the previous 5 years. Analysis was performed using R [14]. Assuming a Poisson distribution to monitor the number of events *λ*_*i* (examinations and fractures) in the fixed interval of time in each of the 5 years 2015–2019 (*n* = 5), we used the maximum likelihood estimator *λ* ® = 1/*n* ∑(*i* = 1)^*n λ*_*i* to estimate the underlying parameter of the Poisson distribution. Conservatively, the lower bound of an asymptotic 95% confidence interval for *λ* ® (= *λ* ®_low) was then used to compute the *p* values to observe as many or fewer events as in 2020.

## Results

Table 1 illustrates the NLP engine’s performance for the concept “fracture affirmed” (RID4650), overall showing an *F*_1_ score of 0.81. The interrater agreement (Cohen’s kappa coefficient [*κ*]) between annotator 1 and annotator 2 was 0.909 in 2019 and 0.854 in 2020, indicating almost perfect agreement [15]. Compared with the consensus reading, the *F*_1_ score of the NLP engine was 0.82 in 2019 and 0.79 in 2020.

**Table 1:** Performance of the NLP engine in the detections of the fractures

	TP	TN	FP	FN	Precision	Recall	*F*_1_ score
Annotator 1—2019	263	606	54	60	0.83	0.81	0.82
Annotator 2—2019	272	593	45	73	0.86	0.79	0.82
Consensus—2019	273	599	44	67	0.86	0.80	0.82
Annotator 1—2020	177	364	55	55	0.76	0.76	0.76
Annotator 2—2020	185	358	48	60	0.79	0.76	0.77
Consensus—2020	194	359	39	59	0.83	0.76	0.79

True positives (TP), true negatives (TN), false positives (FP), false negatives (FN), precision, recall, and *F*_1_ score for the concept “fracture affirmed” (RID4650)

**Fig. 1:**
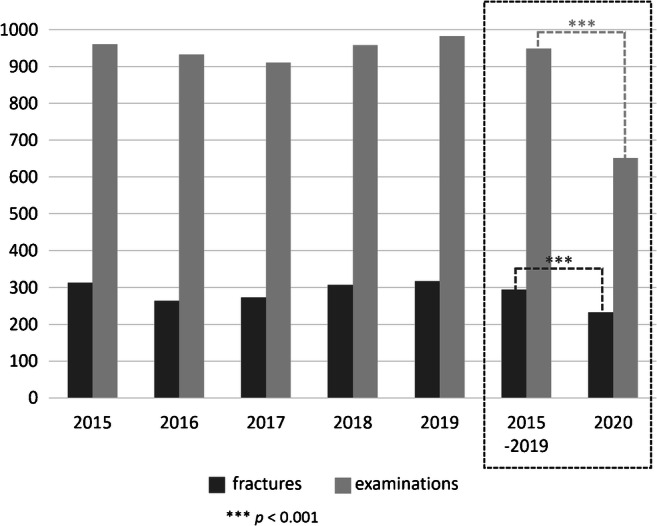
Number of reports with fractures and total number of radiological reports in 2015–2020

Figure 1 illustrates the number of radiological reports with confirmed fractures and the total number of radiological reports analyzed in this study. The average number of fractures in the years 2015–2019 was 294.8 (949.2 examinations), whereas in 2020, the number of fractures was 233 (651 examinations). In 2020, significantly fewer examinations were performed (*p* < 0.001) and significantly fewer fractures were reported (*p* < 0.001), compared with the average of the years 2015–2019.

In 2020, children and adolescents as well as adults up to 65 years presented significantly fewer fractures in significantly fewer examinations compared with the average of the previous 5 years (Table 2). However, the numbers of both examinations and fractures in patients older than 65 years did not change during the COVID-19 pandemic compared with previous years.

**Table 2:** Mean number (*n*) of examinations and fractures in the years 2015–2019 and number of examinations and fractures in 2020 in different age groups

	Mean number in 2015–2019	Conservatively estimated expected number of counts (95 CI)	Number in 2020	*p* value
In total				
Examinations	949	873–972	651	< 0.001
Fractures	295	253–308	233	0.002
< 18 years				
Examinations	231	193–242	93	< 0.001
Fractures	81	60–88	41	< 0.001
18–65 years				
Examinations	526	469–543	380	< 0.001
Fractures	140	111–148	110	0.047
> 65 years				
Examinations	193	159–203	178	0.972
Fractures	74	53–80	82	0.442

The numbers of wrist/hand fractures and elbow fractures were significantly lower in 2020 compared with the average of the years 2015–2019 (Table 3). The numbers of examinations of ankles and knees were significantly lower in 2020, whereas the numbers of ankle and knee fractures did not differ between the average of the years 2015–2019 and 2020.

**Table 3:** Mean number (*n*) of examinations and fractures in the years 2015–2019 and the number of examinations and fractures in 2020 in six investigated anatomical regions

	Mean number in 2015–2019	Conservatively estimated expected number of counts (95 CI)	Number in 2020	*p* value
Hand/wrist				
Examinations	293	251–305	199	< 0.001
Fractures	139	109–147	90	< 0.001
Elbow				
Examinations	99	75–106	68	< 0.001
Fractures	36	22–40	18	< 0.001
Shoulder				
Examinations	110	85–118	97	0.11
Fractures	37	23–41	39	0.67
Ankle				
Examinations	189	156–199	94	< 0.001
Fractures	37	22–41	38	0.63
Knee				
Examinations	154	124–163	93	< 0.001
Fractures	12	4–14	8	0.17
Pelvis/hip				
Examinations	104	79–112	100	0.36
Fractures	36	22–40	40	0.80

## Discussion

In this study, we present the ability of NLP in analyzing radiology reports to identify relevant changes over time in the number of pathologies. More than 5000 reports were automatically categorized into fracture or no fracture. Thus, we were able to study the implications of the COVID-19 pandemic on the incidence of fractures compared with the previous 5 years. We were able to demonstrate that the COVID-19 pandemic led to a significant decrease of fractures in general, especially in children and adolescents, and in adults up to 65 years. To our knowledge, we are the first to use NLP to analyze temporal fluctuations in the frequency of pathologies.

Regarding fractures, the results of our study are in line with previous studies that investigated the influence on the COVID-19 pandemic on upper limb, lower limb, or pelvis fractures [2, 3]. Compared with previous years, in general, there was a decrease in inpatient admission in German orthopedic and trauma surgery clinics [2, 4, 16–20]. In Paris, fewer consultations for joint injuries and fractures of the hand and upper limb were reported during the COVID-19 outbreak, compared with 2019 [3]. In London, referrals for injuries of the upper and lower limb declined in 2020 compared with 2019, whereas the number of hip injuries did not change during the COVID-19 pandemic [4]. This is in line with the results of our study. We did not find a difference between the average in the years 2015–2019 and 2020 when comparing the number of pelvis/hip fractures. In China, most fractures during the COVID-19 pandemic occurred at home and in elderly patients [16]. These patients suffer fractures due to low-energy trauma, often at home or at care homes, and therefore also during a nationwide curfew.

In London, the number of acute pediatric trauma referrals declined significantly in 2020 during the COVID-19 pandemic, in contrast with 2019 [17]. Our results reveal that the number of radiographic studies and the number of fractures in patients under 18 years significantly declined in 2020, whereas the number of fractures in patients older than 65 years remained unchanged. Patients with hip fractures during the COVID-19 pandemic were more comorbid and less active than patients prior to the COVID pandemic [18].

Compared with previous studies [4, 19, 20], we did not find a decline in the number of ankle fractures during the COVID-19 pandemic. Nevertheless, we were able to show significant decreases in the numbers of ankle radiographs and knee radiographs.

NLP has been used before to detect fractures cited in radiological reports or electronic health-care records [9, 21, 22]. Our study analyzed more than 5000 radiological reports concerning various body parts and among all ages. The NLP engine used in the present study achieved an *F*_1_ score of 0.81, slightly lower than the score of just over 0.9 reported in a previous study [21]. Compared with previous studies, we included reports from patients of all ages and several fracture locations, from hand to ankle [21]. It might be more difficult to analyze the presence of fractures in multiple joints and different groups of ages, as the words and phrases of the radiologists probably vary in the different anatomical regions and different fracture types. Furthermore, we analyzed a considerably larger number of radiological reports.

Not all radiological reports can be clearly categorized, even using relatively simple classifications such as fracture affirmed: “yes” or “no.” To define a gold standard, a board-certified radiologist served as a third reviewer and checked those cases in which the results of annotator 1 and annotator 2 did not match (Cohen’s kappa > 0.85). These results were used to measure the NLP performance.

This study has several limitations. First, the content recognition of the pre-trained commercially available NLP engine used in this work is not perfect. With more training and more manual input, it may be possible to further increase the *F*_1_ score. Currently, we are improving the NLP recognition and implementing periodic automated evaluation.

We used the NLP engine to categorize each of the 5379 reports. In this way, we avoided systematic errors that can occur when reports are analyzed by several annotators. Even a single annotator may interpret reports differently from one day to another. Second, we only searched for confirmed fractures. We did not analyze the reports for suspected or negated fractures. In these cases, the interpretation is much more difficult, because in a particular radiological report, there may be coexisting confirmed and excluded fractures at different anatomical locations. Last but not the least, fractures after surgery or during conservative therapy were described in some of the radiological reports. Thus, a certain number of the reports may have been follow-up studies. Since we analyzed every year in the same way, this bias seems likely to be negligible.

In this study, we were able to demonstrate that NLP is capable of automatically identifying relevant changes in the incidence of pathologies. As a use case, we have demonstrated this potential for fracture detection in radiological reports before and during the COVID-19 pandemic. This approach can easily be transferred to other pathologies and other examinations in order to gather epidemiologic information. Furthermore, our approach can also be used to automatically visualize temporal fluctuations in the incidence of pathologies in real time or may trigger root cause analysis.
